# Conservation through the lens of (mal)adaptation: Concepts and meta‐analysis

**DOI:** 10.1111/eva.12791

**Published:** 2019-04-06

**Authors:** Alison Margaret Derry, Dylan J. Fraser, Steven P. Brady, Louis Astorg, Elizabeth R. Lawrence, Gillian K. Martin, Jean‐Michel Matte, Jorge Octavio Negrín Dastis, Antoine Paccard, Rowan D. H. Barrett, Lauren J. Chapman, Jeffrey E. Lane, Chase G. Ballas, Marissa Close, Erika Crispo

**Affiliations:** ^1^ Département des sciences biologiques Université du Québec à Montréal Montreal Quebec Canada; ^2^ Quebec Center for Biodiversity Science Montreal Quebec Canada; ^3^ Biology Department Concordia University Montreal Quebec Canada; ^4^ Biology Department Southern Connecticut State University New Haven Connecticut; ^5^ Redpath Museum and Department of Biology McGill University Montreal Quebec Canada; ^6^ Department of Biology University of Saskatchewan Saskatoon Saskatchewan Canada; ^7^ Department of Biology Pace University New York New York

**Keywords:** adaptation, demographic rescue, evolutionary rescue, gene flow, genetic rescue, hybridization, transgenerational plasticity, translocation

## Abstract

Evolutionary approaches are gaining popularity in conservation science, with diverse strategies applied in efforts to support adaptive population outcomes. Yet conservation strategies differ in the type of adaptive outcomes they promote as conservation goals. For instance, strategies based on genetic or demographic rescue implicitly target adaptive population *states* whereas strategies utilizing transgenerational plasticity or evolutionary rescue implicitly target adaptive *processes*. These two goals are somewhat polar: *adaptive state* strategies optimize current population fitness, which should reduce phenotypic and/or genetic variance, reducing adaptability in changing or uncertain environments; *adaptive process* strategies increase genetic variance, causing maladaptation in the short term, but increase adaptability over the long term. Maladaptation refers to suboptimal population fitness, adaptation refers to optimal population fitness, and (mal)adaptation refers to the continuum of fitness variation from maladaptation to adaptation. Here, we present a conceptual classification for conservation that implicitly considers (mal)adaptation in the short‐term and long‐term outcomes of conservation strategies. We describe cases of how (mal)adaptation is implicated in traditional conservation strategies, as well as strategies that have potential as a conservation tool but are relatively underutilized. We use a meta‐analysis of a small number of available studies to evaluate whether the different conservation strategies employed are better suited toward increasing population fitness across multiple generations. We found weakly increasing adaptation over time for transgenerational plasticity, genetic rescue, and evolutionary rescue. Demographic rescue was generally maladaptive, both immediately after conservation intervention and after several generations. Interspecific hybridization was adaptive only in the F_1_ generation, but then rapidly leads to maladaptation. Management decisions that are made to support the process of adaptation must adequately account for (mal)adaptation as a potential outcome and even as a tool to bolster adaptive capacity to changing conditions.

## 1 INTRODUCTION

Evolutionary concepts are now applied routinely in conservation (Table 1) and crucial to achieving success in many conservation situations (Carroll et al., 2014; Edmands, 2007; Hendry et al., 2011; Stockwell, Hendry, & Kinnison, 2003; Weeks et al., 2011). Commonly, the infusion of evolutionary thinking into species conservation focuses on how phenotypic and/or genetic changes induced by human activities or interventions affect wild population recovery or persistence and, increasingly, community composition and ecosystem function (Bowlby & Gibson, 2011; Dunlop, Eikeset, & Stenseth, 2015; Palkovacs, Moritsch, Contolini, & Pelletier, 2018; Raffard, Santoul, Cucherousset, & Blanchet, 2019). Examples include reintroduction and supplementation programs that use genetically diverse stocks or mimic natural rearing conditions to improve adaptive capacity and minimize adaptation to captivity (Araki, Cooper, & Blouin, 2007; Houde, Garner, & Neff, 2015; Lesica & Allendorf, 1999); commercial aquaculture systems that attempt to limit gene flow between domesticated escapees and their wild counterparts (Castellani et al., 2018; Hindar, Fleming, McGinnity, & Diserud, 2006); and fish/wildlife management, where harvesting strategies can reduce evolution of life‐history trait values that counter those desirable for harvest (Kuparinen & Festa‐Bianchet, 2017).

**Table 1:** Examples of evolutionary principles applied to various conservation strategies

Conservation context	Evolutionary application and goal	References
Management of small, endangered populations	Genetic rescue from inbreeding depression through outbreeding	Westemeier et al. (1998); Pimm, Dollar, and Bass (2017); Frankham (2015)
	Evolutionary rescue via standing or de novo genetic variation	
Captive breeding programs	Minimizing of rapid adaptation to captivity	Fraser (2008); Bowlby and Gibson (2011); Christie et al. (2012)
Demographic rescue		
Reintroduction programs	Adaptive matching of source populations	Lesica and Allendorf (1999); Houde et al. (2015)
Interactions between domesticated and wild species	Mitigating gene flow between domesticated escapees and wild populations	Hindar et al. (2006); Hutchings and Fraser (2008)
Sustainable harvesting, populations	Reducing selectivity (e.g., harvesting of faster growing, later maturing individuals) to avoid undesirable genetic changes to various traits	Heino et al. (2015); Kuparinen and Festa‐Bianchet (2017)
Sustainable harvesting, ecosystems	Reducing selectivity in harvesting to reduce undesirable changes to trophic cascades, communities and ecosystems	Palkovacs et al. (2018)
Endangered species legislation, and designation of conservation units below the species level	Conserving populations harboring unique adaptive characteristics to increase species’ evolutionary potential	Waples (1995); Funk et al. (2012)
Species climate change adaptation	Identifying traits which facilitate or limit adaptive responses to climate change	Donelson, Wong, Booth, and Munday (2016); Schunter et al. (2018)
	Determining the significance of transgenerational plasticity for responses to climate change	

Note

**Figure 1:**
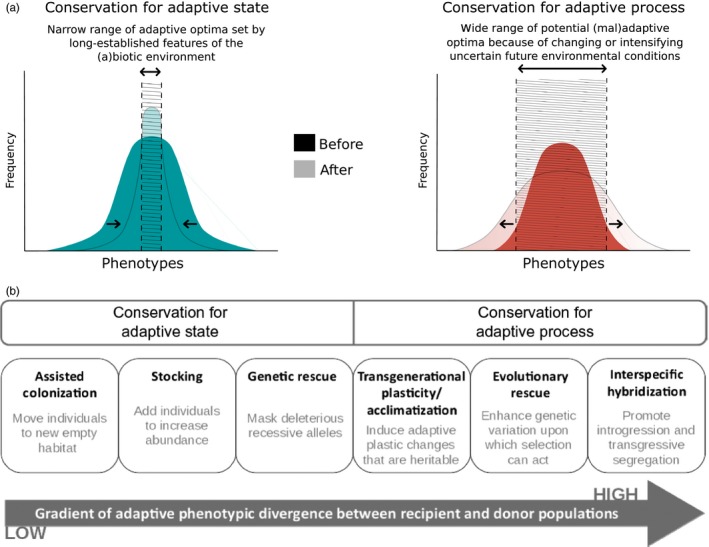
A conceptual classification for considering conservation goals that seek to reduce or integrate (mal)adaptation. (a) Adaptive state versus adaptive process. In both panels, the darker and lighter shading indicates the population trait or fitness frequency before and after implementing a conservation practice, respectively. *Adaptive state* assumes that the population is replenished with individuals so that its fitness returns to a known adaptive optimum presumably set by some long‐established features of the (a)biotic environment. This is illustrated by a narrow range of possible adaptive optima along the phenotype axis in the hatched area of “after” histogram. The result is the mean population fitness closely matches the optimal phenotype, at a given time point, at the expense of reduced heritable trait variation. *Adaptive process*, by contrast, assumes that the optimal phenotype in the future is uncertain because (i) there are multiple (mal)adaptive optima to which it is unknown the population will evolve into the future, or (ii) that a sustained adaptive process will be required to a reach a new optimum in the presence of an intensifying stressor, which may be far from any known phenotype. This is illustrated by the broad range of possible (mal)adaptive optima along the phenotype axis in the hatched area of the “after” histogram. The result is that the heritable trait variation is increased at the expense of reduced mean population fitness in relation to the optimal phenotype. (b) Examples of conservation strategies that occur along a continuum of conservation goals between adaptive state and process. Whereas adaptive state conservation strategies involve the admixture of adaptively similar populations to minimize maladaptation and optimize mean population fitness, adaptive process conservation strategies involve the admixture of adaptively divergent populations to increase heritable (mal)adaptive variation

Some conservation strategies focus more on adaptive state and others more on adaptive process (Figure 1b), though these goals are not mutually exclusive in many instances.

Though evolutionary concepts are now more common in conservation, efforts and theory typically focus on fostering adaptive outcomes without necessarily considering maladaptation. This focus on adaptive outcomes is not unique to conservation, but rather follows a similar pattern seen in basic evolutionary biology, where studies of maladaptation are relatively rare compared to studies of adaptation. Yet maladaptation is common in the natural world, even in circumstances where we expect to find adaptation (Hendry & Gonzalez, 2008; Hereford, 2009; Leimu & Fischer, 2008). Examples of maladaptation include evolutionary traps (Robertson & Chalfoun, 2016), inbreeding depression (Frankham, 2015), and environmental (abiotic or biotic) mismatch of traits (Zimova, Scott Mills, & Nowak, 2016).

But what is maladaptation, adaptation, and (mal)adaptation? Maladaptation has multiple descriptions and therefore requires specific language (Crespi, 2000). However, in all cases, maladaptation refers to a condition of suboptimal fitness. Here our focus is on maladaptation at the population level, both in absolute and relative fitness terms. For a population, *absolute maladaptation* occurs whenever population mean fitness is below the rate of replacement, and the population is in a state of decline. In discrete time, absolute maladaptation can be expressed as $\overset{¯}{W}<1\overset{¯}{W}<1\overset{¯}{W}<1$ and is equivalent to *r* < 0 instantaneous time (as often expressed in ecological models of population growth). Thus, a population is absolutely maladapted whenever $\overset{¯}{W}<1\overset{¯}{W}<1\overset{¯}{W}<1$ or absolutely adapted whenever $\overset{¯}{W}<1\overset{¯}{W}<1\overset{¯}{W}<1$. *Relative maladaptation* ($\overset{¯}{w}\overset{¯}{w}\overset{¯}{w}$occurs whenever the absolute fitness, $\overset{¯}{W}\overset{¯}{W}\overset{¯}{W}$, is less than the fitness of some reference population (e.g., the fitness of the most‐fit population within a metapopulation). For instance, if population A has mean absolute fitness of ${\overset{¯}{W}}_{A}=1.0{\overset{¯}{W}}_{A}=1.0{\overset{¯}{W}}_{A}=1.0$ and population B has mean absolute fitness of ${\overset{¯}{W}}_{B}=0.80{\overset{¯}{W}}_{B}=0.80{\overset{¯}{W}}_{B}=0.80$, population B is maladapted relative to population A (${\overset{¯}{w}}_{B}={\overset{¯}{W}}_{B}/{\overset{¯}{W}}_{A}\;=\;0.8{\overset{¯}{w}}_{B}={\overset{¯}{W}}_{B}/{\overset{¯}{W}}_{A}\;=\;0.8{\overset{¯}{w}}_{B}={\overset{¯}{W}}_{B}/{\overset{¯}{W}}_{A}\;=\;0.8$). Thus, a population is relatively maladapted whenever $\overset{¯}{w}<1\overset{¯}{w}<1\overset{¯}{w}<1$. These definitions of maladaptation can also apply to an individual or a trait value in terms of their absolute and relative fitness. For both absolute or relative measures of fitness, maladaptation refers to $\overset{¯}{W}\;\text{or}\;\overset{¯}{w}<1\overset{¯}{W}\;\text{or}\;\overset{¯}{w}<1\overset{¯}{W}\;\text{or}\;\overset{¯}{w}<1$, adaptation refers to $\overset{¯}{W}\;\text{or}\;\overset{¯}{w}>1\overset{¯}{W}\;\text{or}\;\overset{¯}{w}>1\overset{¯}{W}\;\text{or}\;\overset{¯}{w}>1$, and (mal) adaptation refers to the continuum of fitness variation from maladaptation to adaptation: $1<\;\overset{¯}{W}\;<\;1\;\text{and}\;1\;<\;\overset{¯}{w}\;<\;11<\;\overset{¯}{W}\;<\;1\;\text{and}\;1\;<\;\overset{¯}{w}\;<\;11<\;\overset{¯}{W}\;<\;1\;\text{and}\;1\;<\;\overset{¯}{w}\;<\;1$. Ultimately, population size, carrying capacity, and temporal dynamics are important parameters for interpreting the severity of maladaptation.

If maladapted populations are prone to population decline over the long run (Hendry & Gonzalez, 2008)—what conservation biologists have been studying for decades—why should we bother with a conceptual classification for conservation that implicitly considers maladaptation in the continuum of fitness responses that range from maladaptive to adaptive? First, maladaptation has numerous causes beyond habitat loss and exploitation that can act jointly, and an improved understanding of the underlying mechanisms of maladaptation should help inform the chosen conservation strategy. Second, maladaptation can evolve over time (even resulting from management actions aiming to improve population success), exacerbating fitness declines. Third, maladaptation is more likely to occur as human activities accelerate the rate of environmental change and increase environmental novelty. Current global species extinction rates are estimated to be ~1,000 times the predicted background rate (Pimm et al., 2014), reinforcing the idea that maladaptation is a common outcome of human‐induced environmental change. Such environmental change may constrain local adaptation if populations become too small (thus accelerating genetic drift or inbreeding), lack standing adaptive genetic variation, and/or have had insufficient time to adapt to abrupt changes (Gonzalez, Ronce, Ferriere, & Hochberg, 2013; Rolshausen et al., 2015). Fourth, our understanding of the likelihood of success of various evolutionary conservation strategies is incomplete in terms of their specific abilities to facilitate population persistence while lessening maladaptation. Overall, understanding the root causes of maladaptation can inform decisions regarding conservation strategies, while appreciating the dynamics of (mal)adaptation can inform conservation goals.

### 1.1 Conserving for adaptive states versus adaptive processes

The realization that maladaptive fitness variation is characteristic of many natural and unthreatened populations (Fraser, Weir, Bernatchez, Hansen, & Taylor, 2011; Hendry & Gonzalez, 2008; Hereford, 2009) suggests that traditional conservation ideologies focused on eliminating maladaptation might benefit from a more holistic view of (mal)adaptive dynamics and (mal)adaptive trait variation (e.g., across fitness landscapes). The prevailing conservation ideology of conserving populations in an *adaptive state* focuses on mitigating extinction risk by reducing phenotype–environment mismatch (Weeks et al., 2011). Such *conservation for an adaptive state* assumes that any factor that increases absolute or relative maladaptation in a population can have negative consequences for either short‐term or long‐term persistence (Edmands, 2007; Frankham, 2015). In other words, the goal of conservation for adaptive state is to keep the focal population trait mean as close to that of the optimum phenotype, while minimizing (mal) adaptive fitness variation (Figure 1a). Nevertheless, apparently maladapted wild populations may persist in some situations if maladaptation is transient, sustained by bet‐hedging strategies (Simons, 2009), or offset by immigration (Gonzalez & Holt, 2002; Holt, Pickard, & Prather, 2004; Negrín Dastis & Derry, 2016) if enemies are excluded allowing persistence of maladapted phenotypes (Rolshausen et al., 2015); if individuals collect in poor habitats (Brady, 2013); and even if populations have small effective population sizes (*N*_e_) and/or substantial genetic load over the long term (Benazzo et al., 2017; Perrier, Ferchaud, Sirois, Thibault, & Bernatchez, 2017). Moreover, while intra‐ or interspecific hybridization can generate phenotypic–environment mismatch, it is rarely linked to widespread extinction (Todesco et al., 2016). Therefore, depending on the dynamics in a system, conserving for an adaptive state might in fact counter the existing dynamics that support population persistence (Broadhurst et al., 2008; Sgro, Lowe, & Hoffmann, 2011; Weeks et al., 2011).

Conservation strategies that focus heavily on preserving an adaptive state (e.g., when defining conservation units below species levels: Funk, McKay, Hohenlohe, & Allendorf, 2012; Waples, 1995) may be inefficient at promoting adaptive potential. When populations are conversely viewed along a gradient of adaptation or of maladaptation, such inefficiencies can be improved. And when it is acknowledged that populations may persist despite having some degree of maladaptation (Leimu & Fischer, 2008), inaction in certain situations may be a more pragmatic conservation approach than action. For example, some small *N*_e_ populations with high genetic load can persist over the long term without need of genetic rescue (Benazzo et al., 2017), a tool often requiring significant conservation resources (Waller, 2015).

A relatively new ideology of *conservation for an adaptive process* asserts that conservation should also maintain or enhance population adaptive potential in the face of environmental change (Ferrière et al., 2004; Gellie, Breed, Thurgate, Kennedy, & Lowe, 2016; Weeks et al., 2011). This idea is similar to the notion that increasing genetic diversity should benefit small populations (e.g., through genetic rescue), but explicitly considers the necessary outcome of some degree of maladaptation at the population level. The goal of conservation for adaptive process is to increase heritable trait variation at the expense of reduced mean population fitness in relation to the optimal phenotype (Figure 1a). Therefore, while conservation for adaptive state predicts that maladaptation will negatively influence population fitness, conservation for adaptive process predicts that maladaptation can positively influence long‐term population fitness under environmental change, despite suboptimal fitness at any given point in time. For instance, hybridization between diverse source populations or even related species can result in maladaptive trait and genetic variation that lowers mean population fitness in any given generation but maintains on average higher fitness over time due to genetic resiliency (Hamilton & Miller, 2016; Todesco et al., 2016; Weeks et al., 2011; but see Kovach, Luikart, Lowe, Boyer, & Muhlfeld, 2016).

### 1.2 The importance of considering (mal)adaptation in conservation

Herein we build on the connection noted by others (Weeks et al., 2011) between conservation goals focused at a given point of time on current population fitness (conservation for an adaptive state) versus maintaining adaptive or evolutionary potential (conservation for an adaptive process). Conservation goals may therefore be visualized as a continuum of adaptive phenotypic divergence between recipient focal populations to conserve, and donor populations from which migrants are drawn (Figure 1a). Although it is instructive to consider their ideological contrasts, conservation for adaptive state versus conservation for adaptive process are not necessarily mutually exclusive when put into practice (Table 1). Rather, different conservation strategies can be placed at different points along the ideological gradient of adaptive state and process (Figure 1b). However, the choice of application often depends more on the state of the focal population to be conserved as well as the state and availability of donor populations than choice of conservation ideology, especially for endangered species.

First, we used case examples to illustrate how conservation that implicates a positive role for (mal)adaptation (Figure 1b) can contribute to improved conservation practices, and how natural processes (e.g., migration load, introgression) and intervention tactics (e.g., hybridization, assisted migration) can result in increased maladaptation in the short term, but, in some cases, may lead to more viable populations in the long term. Second, we conducted a meta‐analysis to test if *adaptive state* versus *adaptive process* conservation goals differ in success between short‐ and long‐time periods, over generations. We hypothesized that adaptive state‐based strategies would tend to increase fitness over short periods of one to two generations, but that they would tend to be less effective than process‐based strategies at increasing fitness over a larger number of generations. We evaluated this hypothesis using fitness data compiled from studies reporting outcomes from a broad spectrum of conservation strategies (Figure 1b).

## 2 EXAMPLE STRATEGIES FOR ADDRESSING MALADAPTATION IN CONSERVATION

Our discussion of several conservation strategies (Table 1 and Figure 1b) illustrates (a) how different causes and states of maladaptation can exist within focal populations, (b) how strategies can be relevant for alleviating maladaptation, and (c) how maladaptation might persist to impact population persistence and/or recovery when maladaptation is not fully considered during implementation. Some untreated strategies are considered elsewhere. For example, under some conditions, harvesting selection and evolution may lead to maladaptation that reduces population growth and recovery even following the cessation of harvesting (Dunlop et al., 2015), but this remains a subject of debate (see Heino, Pauli, & Dieckmann, 2015; Kuparinen & Festa‐Bianchet, 2017). Other emerging topics, such as the use of genomics or biotechnologies to track or overcome maladaptation in wild populations, are treated in Boxes 1 and 2.

Box 1 **Molecular level forecasting of maladaptation**

1 Genomewide sequencing technologies can facilitate the recognition and quantification of putative maladaptation in natural populations via detection of genomic signatures of maladaptation. For instance, the (mal)adaptive state of a population facing harsh environmental shifts can be quantified by the pattern of selective sweeps. While hard selective sweeps would be a strong signature of evolutionary rescue, partial or soft sweeps could demonstrate that a population is potentially (mal)adapted (Agashe, Falk, & Bolnick, 2011). To strengthen this detection, the intensity of selective sweeps could be studied over time and compared to a reference population that shows strong sweeps at loci responsible for adaptation to the same environmental conditions. This type of measurement of maladaptation remains relatively complex as different demographic history, effective population size (*N*_e_), and mutation rate can lead to evolutionary rescue via soft selective sweeps (Wilson, Pennings, & Petrov, 2017). Overall, measures of selective sweeps over multiple generations might be more informative than within generations where genetic structure may confound with actual changes across generations. Nevertheless, such monitoring should be evaluated with specific criteria and trigger points allowing practitioners to decide whether an intervention should be initiated or whether the monitoring program should be adjusted (Flanagan, Forester, Latch, Aitken, & Hoban, 2018). For instance, if a selective sweep reaches a certain intensity (trigger point), practitioners may decide to intervene.

Levels of genetic load can also signal (mal)adaptation. Indeed, reduction in mean fitness across time due to high frequencies of deleterious mutations, as compared to a reference population with high fitness, would be indicative of maladaptation. These measurements would depend on demography as deleterious mutations can be purged in small populations (Perrier et al., 2017), thereby reducing genetic load. Such inferences can also give information about the “lag load” or the degree to which the fitness of local genotypes lags behind the optimal genotypes of a reference, well‐adapted population (Smith, 1976).

Estimating and monitoring *N*_e_ from genomewide markers offers another approach to tracking (mal)adaptation in a population. When combined with knowledge of trait distributions and long‐term fitness landscapes (and thus genetic load), one could eventually decide to apply a conservation strategy if *N*_e_ is low and genetic load is strong. Moreover, effective/census population size ratio estimations (*N*_e_/*N*) calculated across time offer insights about the persistence of populations facing new environmental challenges (Palstra & Fraser, 2012); a small ratio would reflect a more rapid loss of genetic diversity (and perhaps greater risk of becoming maladapted) than from an equal‐sized population with a greater ratio.

Knowledge of the genetic variants responsible for adaptation to environmental variables can also help to forecast putative maladaptation by verifying if populations carry the relevant adaptive alleles to remain viable in a future context. For example, candidate SNPs for climate adaptation were described and used as predictors for (mal)adaptation in the maritime pine *Pinus pinaster* (Jaramillo‐Correa et al., 2014). The authors used genomewide sequences from hundreds of individuals across the maritime pine range and identified SNPs associated with climate. Combined with a common garden experiment under arid and hot conditions, they discovered that the frequency of local alleles correlated with survival. These candidate SNPs were then used to forecast the likely destiny of natural forest ecosystems under climate change scenarios. With such knowledge, practitioners could decide to (a) increase within‐population variation by introducing individuals from populations that have historically encountered a distinct climate and thereby increase the genetic variation available for adaptation, or conversely (b) if resources are limited, triage those populations containing adaptive alleles for conservation protection (assuming that the genomic basis for adaptive responses is unambiguous). The former is a form of assisted gene flow that favors the introgression of specific alleles which will be targeted by selection (Aitken & Whitlock, 2013). This is particularly relevant at species’ range margins such as the Douglas‐fir, where populations from northern distribution boundaries experience rapid changes in environmental conditions (St Clair & Howe, 2007).

Overall, genomewide knowledge of thousands of molecular variants will improve the precision of the parameters used to detect signatures of molecular maladaptation at many orders of magnitude above the traditional number of predesigned genetic markers. In addition, identifying maladaptive loci across the genome will offer strong insights about the genetic basis of maladaptation. This knowledge will allow practitioners to improve their conservation strategies such as assisted gene flow and genetic rescue with a finer evaluation of inbreeding, population size, genetic load, maladaptive loci, and other factors (Díez‐del‐Molino, Sánchez‐Barreiro, Barnes, Gilbert, & Dalén, 2018; Schell, 2018).

Box 2 **Maladaptation concerns in the era of genetic engineering**

1 Evidence has shown that current conservation strategies have failed to slow the rate of biodiversity loss (Tittensor et al., 2014). Whether through back‐breeding, cloning, or assisted conservation applications (Fraser, 2008; Holt et al., 2004; Loi et al., 2001), current indications have shown that conservation practitioners are not able to cope with the speed of environmental changes and species extinction rate. Novel, rapid, technological advances may provide a means of overcoming maladaptation in populations and bringing back extinct species of conservation interest, while balancing of course, ethical concerns.

Synthetic biology and gene drive systems offer a potentially revolutionary solution for the conservation of populations experiencing maladaptation (Piaggio et al., 2017). Indeed, following the diagnosis or forecasting of maladaptation in a population, one could imagine employing genetic engineering to increase the adaptive potential of a threatened population by directly introducing the appropriate genetic variants in its genome (see Box ). This technology has been successfully used in natural populations (e.g., in mosquitoes and rodents), although this research is still in the early stages (Hammond et al., 2016). Although the use of such technologies raises important ethical questions (direct modification of wild species and “responsible stewardship”) (Piaggio et al., 2017), it remains a promising tool for conservation strategies.

Among gene drive technologies, the CRISPR‐Cas9 system represents the most powerful technology for genetic editing, and this has the potential to solve a wide range of conservation issues such as pest management, control of invasive species, and de‐extinction (Shapiro, 2015; Webber, Raghu, & Edwards, 2015). This genetic array has the ability to cut, copy, and paste any genetic information into the targeted genome by suppressing, modifying, adding or removing DNA bases (Jinek et al., 2012). Such technology can be used to spread advantageous traits through populations at a speed far greater than would otherwise be possible by introducing lost or nonexistent adaptive genetic diversity, as part of the emerging field of “synthetic biodiversity” (Champer, Buchman, & Akbari, 2016; Shapiro, 2015).

Other gene drive systems are starting to emerge, but only at the theoretical level. For instance, the use of transposons or B chromosomes has the potential to spread specific genetic variants within natural populations (Champer et al., 2016). The Killer–Rescue system can also help for pest management by inducing a population to crash (Gould, Huang, Legros, & Lloyd, 2008). Research in these latter techniques is still in its infancy and development of the CRISPR array appears to be the most promising tool for maintaining biodiversity (Champer et al., 2016; Shapiro, 2015) and facilitating adaptation (Thomas et al., 2013).

### 2.1 Conservation for an adaptive state

#### 2.1.1 Assisted migration

On the extreme end of the continuum of conserving the adaptive state, (Figure 1b), assisted migration (also referred to as assisted colonization or translocation) is the intentional release of animals in the wild to previously unoccupied ranges to establish or save a threatened population (Armstrong & Seddon, 2008; Griffith, Scott, Carpenter, & Reed, 1989). The goal of this approach is to move organisms to an environment to which they are already adapted, and away from an environment that is no longer suitable due to changing environmental conditions. As such, assisted migration may be a viable strategy to assist threatened, isolated endemics from climate change when insufficient genetic material is present for evolutionary adaptation (Thomas, 2011). However, moving species outside their native range risks they become invasive in their new habitat, potentially reducing local biodiversity, disrupting ecological interactions, spreading pathogens and parasites, and causing other unpredictable ecological impacts (Ricciardi & Simberloff, 2009). Therefore, assisted migration is often viewed as a last resort method to address extinction risk of (primarily) climate imperiled species (Carrete & Tella, 2012). New decision‐making frameworks hold great promise for improving assisted migration (Chauvenet, Ewen, Armstrong, Blackburn, & Pettorelli, 2013; Hällfors, Aikio, & Schulman, 2017). For example, Lunt et al. (2013) suggest prioritization of taxa that are likely to perform important ecological functions after relocation. Nevertheless, such frameworks rarely consider how maladaptation per se is manifested in assisted migrations and under what conditions it is more likely to hinder or facilitate assisted migration.

Maladaptation can occur in translocated populations for different reasons. Populations may be maladapted in habitats to which they are translocated because of conditions that had not been initially assessed when determining habitat suitability, or because they have a small, initial population size that leaves them more vulnerable to genetic drift and deleterious mutations, which can drag population trait values away from local optimums (maladaptation through “inaccuracy”). To prevent such issues, multiple translocations into the new environment can be used to replenish genetic diversity within the population, especially if translocated populations remain small (Armstrong & Seddon, 2008; Griffith et al., 1989). However, maladaptation might be density dependent and thus persist in a translocated population if too many individuals occupy the new environment's fitness peak (maladaptation through “too many darts”). For example, even if the translocated population trait value perfectly matches the new environment's fitness peak, individual fitness will decrease because the environment's carrying capacity is too low for the increased number of organisms following the translocation event. In the case of the Seychelles Warbler (*Acrocephalus sechellensis*), as an example, this density‐dependent reduction in fitness occurs in the form of decreased reproductive output following translocation (Brouwer et al., 2009).

Assisted migration should be considered when population maladaptation indicates a need for intervention, and the goal of management is to limit or eliminate maladaptation (i.e., conservation for adaptive state in the translocation environment). But in a changing environment, a lack of maladaptation in the population—that is, a limited variety of available phenotypes—can potentially cause further problems, and so conservation for adaptive process is also important. Too little phenotypic variation around the fitness optimum in a population facing environmental change (shifting the fitness optimum) can decrease future adaptive potential. We expect that for a given environment, there should be an optimal amount of variation around the fitness optimum (maladaptation through “imprecision”) that would facilitate future adaptation and yet not reduce mean fitness so much that the population cannot persist. While studies on assisted migration often focus on determining what range of environmental conditions will promote individual survival if translocation was to be used as a conservation measure (Roncal, Maschinski, Schaffer, Gutierrez, & Walters, 2012; Tabi, Campo, Aguado, & Mulet, 2016) and, to a lesser extent, the impact management has on subsequent genetic diversity (Komdeur, Kappe, & Zande, 1998), we are not aware of any studies investigating what level of population variation is required to promote long‐term population persistence.

#### 2.1.2 Demographic rescue

Captive breeding and supplementation programs aim to increase the abundance of wild populations, and often (but not always) ignore adaptation and adaptive similarity between recipient and donor populations. This strategy is used when populations are in severe decline and at risk of imminent extirpation, or that are being recovered as part of species restoration efforts (Naish et al., 2007; Snyder et al., 1996). Yet captivity routinely causes plastic and/or genetic changes to traits that are associated with fitness in the wild, because selective pressures vary dramatically between captive and wild environments (Araki et al., 2007; Fraser, 2008; Johnsson, Brockmark, & Näslund, 2014). Consequently, individuals released back into the wild are often maladapted due to trait mismatch between the captive‐reared phenotype and the natural habitat (Johnsson et al., 2014; Roberts, Taylor, & Leaniz, 2011), often significantly reducing the likelihood of enhancing the viability of wild populations (Bowlby & Gibson, 2011; Satake & Araki, 2012; Willoughby & Christie, 2018). Such maladaptation can be manifested quickly in one or two generations of captive exposure (Araki et al., 2007; Christie, Marine, French, & Blouin, 2012) and vary substantially among populations brought into captivity (Fraser et al., 2019).

The mechanisms and conservation implications of such maladaptation have been most widely considered in socioeconomically important salmonids (Clarke, Fraser, & Purchase, 2016; Johnsson et al., 2014). These animals are reared in hatcheries by the billions annually and released into nature as part of many conservation programs (Naish et al., 2007). Despite quantitative advances to understanding how to achieve demographic benefits of captive rearing/breeding while minimizing maladaptation in the wild (Bowlby & Gibson, 2011; Satake & Araki, 2012), the full long‐term consequences of maladaptation induced in captivity on postrelease fitness have not been well studied empirically (Johnsson et al., 2014; Willoughby & Christie, 2018).

Encouragingly, some captive‐induced maladaptation might be overcome quite quickly in the wild. Despite trait‐mismatch, captive‐reared and wild individuals commonly interbreed, rendering populations a mix of captive‐wild hybrids (Hansen, 2002). These hybridized populations often persist after supplementation at “normal” densities, suggesting that natural selection removes maladaptive alleles favored in the captive environment after hybridization with wild individuals in nature, returning fitness of wild individuals to previous levels in as few as 6–11 generations (Harbicht, Wilson, & Fraser, 2014). Furthermore, the establishment of feral populations from domesticated captive strains provides indirect evidence that maladaptive changes resulting from captive exposure can be overcome in some situations.

Collectively, a main implication for conservationists is that pinning down the nature and extent of maladaptation in captive breeding and supplementation programs will (a) help to achieve desired effects of programs intended to demographically rescue wild populations; (b) shed further light on how wild populations can persist with some maladaptation, and for how long, and finally (c) facilitate improved interpretations of fitness variation on population growth in the natural environment.

#### 2.1.3 Genetic rescue

Classic theory suggests that habitat fragmentation and loss will reduce population size, decrease dispersal rates, and induce an extinction vortex. The reduction of genetic diversity that occurs in an isolated population of small *N*_e_ leads to increased maladaptation through increased inbreeding and drift, further reductions of genetic diversity, poorer offspring viability and recruitment, and even smaller *N*_e_, which enhances extinction risk (Soulé & Simberloff, 1986). Genetic rescue, the infusion of genetic material from donor populations to overcome such maladaptation, is increasingly adopted for managing highly inbred wild populations that have experienced rapid declines (Frankham, 2015; Hedrick, Adams, & Vucetich, 2011; Tallmon, Luikart, & Waples, 2004).

Numerous success stories exist for genetic rescue efforts, some classic cases including the Florida panther (*Puma concolor*; Johnson et al., 2010), greater prairie chickens (*Tympanuchus cupio pinnatus*; Westemeier et al., 1998), and adders (*Vipera berus*; Madsen, Ujvari, & Olsson, 2004). In each of these three cases, the addition of conspecific donors (not necessarily of the same subspecies) reversed trends of population declines and enhanced recruitment above and beyond any changes in abundance due directly to the addition of individuals to the population. Common characteristics of the outbred populations, relative to inbred populations, included enhanced reproductive success and measurable increases in genetic diversity following several generations post genetic rescue.

While genetic rescue has been effective in many empirical cases (Frankham, 2015), maladaptation can persist after genetic rescue attempts due to resistance of the recipient population to donor individuals or maladapted donors. For example, local adaptation leads to higher fitness of resident phenotypes relative to donor phenotypes, so donor genotypes can be relatively maladapted to the local environment of the recipient population. Genetic rescue attempts might also be unsuccessful when mating behaviors or the phenology of donor and recipient populations are mismatched, or when genomic incompatibilities occur.

Donor individuals may also have low absolute fitness for several reasons, leading to resistance to establishment of donor alleles in the recipient population. Donors may be inbred, have low genetic variability, be too old to reproduce, or be in poor condition; or perhaps too small a number of donors are used in conservation efforts (Zeisset & Beebee, 2013). The success of donor individuals and their effects on population recovery may also depend on an interaction between sex and condition (Linklater, 2003; Zajitschek, Zajitschek, & Brooks, 2009). Hence, managers must carefully consider which individuals would be best suited for specific cases. Of course, maladaptation might persist because the environment is changing, in which case selection would continuously act to reduce the population size. Strategies that enhance the adaptive process, and they can afford organisms or populations the ability to change in response to the environment, might be superior in these cases.

### 2.2 Conservation for an adaptive process

#### 2.2.1 Inducing transgenerational acclimatization/plasticity

To reduce species’ maladaptation in the face of environmental change, conservation programs are beginning to incorporate acclimatization plans. Such acclimatization involves phenotypically plastic responses in physiology, morphology, or behavior that can help maintain fitness in novel environments (Angilletta, 2009; Donelson, Salinas, Munday, & Shama, 2018; Narum, Campbell, Meyer, Miller, & Hardy, 2013). In contrast to genetic adaptation, acclimatization can occur as an immediate response to new environmental conditions within a single generation. While most acclimatization studies involve within‐generation plasticity, transgenerational plasticity (TGP) can also occur when the environment experienced by parents shapes trait reaction norms of their offspring (Salinas & Munch, 2012; Schunter et al., 2018; Veilleux et al., 2015). Such TGP is any effect on the offspring phenotype brought about by the transmission of factors other than DNA sequences (e.g., nutritional, somatic, cytoplasmic) from parents or grandparents (Bonduriansky & Day, 2009). Of course, there are several nongenetically inherited factors that are not easily distinguished or mutually exclusive from TGP, including maternal effects (Shama et al., 2016), epigenetic inheritance (Klironomos, Berg, & Collins, 2013), and genomic imprinting (Bartolomei & Ferguson‐Smith, 2011). The main point is that TGP can either enhance population viability in the face of environmental change by increasing the likelihood of a phenotype–environmental match in offspring (Miller, Watson, Donelson, McCormick, & Munday, 2012), or decrease offspring viability if, for example, offspring disperse away from the maternal environment (Moran, Dias, & Marshall, 2010).

Maladaptation can be also induced through TGP, indicating that the benefits of TGP are context‐dependent (Marshall, 2008) or may only partially compensate for negative effects induced by stress exposure. For example, Allan, Miller, McCormick, Domenici, and Munday (2014) tested the effects of acute CO_2_ exposure on the escape response of a juvenile fish whose parents had been reared in either control or high CO_2_ environments. Acute exposure to elevated CO_2_ had negative effects on both juvenile responsiveness and locomotor performance. Parental exposure to high CO_2_ reduces these effects on some traits, but it did not completely compensate for negative effects of CO_2_ exposure on escape response (Welch, Watson, Welsh, McCormick, & Munday, 2014). There may also be a trade‐off across life‐history stages, with enhanced performance from TGP in one life stage having negative effects on other life stages, an outcome that may be common in marine organisms with dispersive larvae (Marshall & Morgan, 2011).

Collectively, parental exposure to stressors may be important in facilitating persistence of organisms in the face of rapid environmental changes. Yet, carry‐over effects may also have negative consequences when environmental shifts occur between generations or life stages. Inclusion of maladaptive considerations in TGP research will specifically enrich its application as a conservation tool because nongenetic parental effects may alter adaptive responses to elevated environmental stress. While TGP may play an important role in modifying the impacts of global change, these effects and not uniformly positive (Guillaume, Monro, & Marshall, 2016), but such responses can be adaptive when the parental environment is a good predictor of the offspring environment (Burgess & Marshall, 2014).

#### 2.2.2 Evolutionary rescue

While genetic rescue has been well documented in isolated, inbred populations, less studied is how the infusion of alleles may be beneficial for evolution in response to environmental change, that is, evolutionary rescue (Carlson, Cunningham, & Westley, 2014; Gonzalez et al., 2013). Enhancing genetic variation might benefit wild populations living in stressful environments, or in situations where genetic rescue may be deemed unnecessary in the short term.

Empirical examples of evolutionary rescue in conservation contexts are rare. The best documented cases are from observational studies on adaptation of vertebrate pest populations (rats and rabbits) to control agents (reviewed by Vander Wal, Garant, Festa‐Bianchet, & Pelletier, 2013) and in experimental laboratory studies. In many of the experimental studies, the ability to evolve in response to an imposed stressor was related to population size in the absence of immigration (Bell & Gonzalez, 2009; Willi & Hoffmann, 2009). Some more recent studies have examined whether manipulating immigration can infuse adaptive alleles into a population, speeding evolutionary rescue. In a study on the evolutionary response to an insecticide in flour beetles (*Tribolium castaneum*), it was found that survival in the presence of the insecticide was greater after *high* than *low* migration from a population carrying an allele for insecticide resistance, although neither experimental treatment resulted in fixation of the resistance allele after seven generations (Rafter, McCulloch, Daglish, & Walter, 2017). Also using flour beetles in an experimental setup, evolutionary rescue was shown to reduce extinction risk, and extinction risk was inversely related to population size (Hufbauer et al., 2015).

Of course, maladaptation might persist because the environment is continuously changing. In this case, selection would continuously act to reduce the population size, even when evolutionary rescue is attempted by managers or occurring naturally due to standing variation, because evolutionary adaptation inherently cannot occur without reducing population size. Evolutionary rescue might help in cases of environmental change if donors were very genetically diverse, but if environmental changes are large and sustained, populations will continue to be maladapted to some extent because fitness can never be as high under shifting conditions as in a constant environment due to the selection pressures imposed on the population.

#### 2.2.3 Interspecific hybridization

Increasing rates of hybridization may generate substantial maladaptation and result in the extinction of unique populations or species through unsuccessful reproductive effort or via introgression with a more common species (Kovach et al., 2016; Vilà, Weber, & Antonio, 2000; but see Todesco et al., 2016). For example, hybridization between wild salmon and recurrent farmed escapees from aquaculture has occurred widely (Glover et al., 2012). Domestication of farmed salmon renders them maladapted in the wild, so farmed‐wild hybridization erodes wild fitness and can have long‐term, detrimental consequences for the life history, genetic characteristics, and viability of wild salmon populations (Bolstad et al., 2017; Glover et al., 2017).

Hybridization, however, is also a major source of evolutionary innovation. It offers the opportunity for phenotypic and genetic novelty at a pace much faster than within‐species adaptation (Hamilton & Miller, 2016; Pfennig, Kelly, & Pierce, 2016). This is because adaptive genetic variance can be rapidly increased through adaptive introgression (Hamilton & Miller, 2016; Song et al., 2011; Stelkens, Brockhurst, Hurst, & Greig, 2014). Moreover, one of the few examples of experimental work involving advanced generation hybrids (F_14_) showed that hybrid breakdown of fitness in F_2_ or F_3_ generations following F_1_ heterosis can be recovered in subsequent generations, especially under environmental stress (Hwang, Pritchard, & Edmands, 2016). Finally, interspecific hybridization can result in transgressive segregation, the appearance of extreme phenotypes in F_2_, backcrossed, or advanced generation hybrids, and this can facilitate niche transitions or create new ecological opportunity (Pereira, Barreto, & Burton, 2014; Rieseberg, Archer, & Wayne, 1999).

Many of the extreme phenotypes generated through adaptive introgression may be maladapted under current conditions but have the potential to enhance the adaptability of threatened populations facing rapid environmental change (Baskett & Gomulkiewicz, 2011; Hamilton & Miller, 2016). Indeed, adaptive introgression would increase intraspecific trait variance on which selection could act, at the potential cost of creating transient maladaptive population trait means because not all trait variance introduced would be adaptive at a given point in time (adaptive process; Figure 1b). Theoretically, adaptive introgression can reduce population decline in response to a stressor and accelerate population rebound during the U‐shaped trajectory beyond what would be expected from standing within‐population genetic variation alone (Carlson et al., 2014; Hamilton & Miller, 2016). While conditions that allow evolutionary rescue to occur via adaptive introgression have been explored in laboratory conditions (Stelkens et al., 2014) and with mathematical models (Baskett & Gomulkiewicz, 2011), evolutionary rescue has been demonstrated only a few times in nature and conditions under which it hinders or facilitates population recovery merits further study (Carlson et al., 2014; Hamilton & Miller, 2016).

## 3 QUANTITATIVE SYNTHESIS OF MALADAPTATION IN EXISTING CONSERVATION STRATEGIES

In conservation, relative fitness can be drawn from inferences between populations (conservation inaction vs. conservation action) and absolute fitness can be drawn from inferences within the same population (before and after the conservation action). We expect, when conserving for the adaptive *state*, that relative or absolute fitness will be greater when implementing a conservation intervention on the *short* term (e.g., one to two generations); when conserving the adaptive *process*, relative or absolute fitness will be greater when implementing a conservation intervention on the *long* term (many generations).

### 3.1 Literature review and criteria for quantitative synthesis

We performed a literature search for primary journal articles using Web of Science or Google Scholar separately for each of the conservation strategies/themes highlighted in Figure 1b. Note that some of these conservation strategies focus more on adaptive state, others on adaptive process, though these goals are not mutually exclusive in many instances (Table 1). Paper relevance was first assessed by reviewing titles and abstracts, after which we attempted to extract data.

To determine whether an article had usable data on maladaptation, we used the following four criteria: (a) data must include a fitness metric, including survival, fecundity or egg/seed size, or abundance or recruitment; correlates of fitness such as growth or body size were rejected; (b) either data from a control without a conservation intervention (to measure relative fitness), or data from the population before conservation was implemented (to measure absolute fitness), must be available; (c) data from at least two different time periods after the conservation intervention must be available; (d) sample size, and a metric of sampling variance (standard error or deviation, or confidence intervals) for the fitness metrics must be reported. A second dataset included all these studies, plus studies that met only the first three criteria, so that we could employ a test with a larger dataset based on categorical data (fitness improved, declined, or did not change) instead of analyzing effect sizes.

The following keywords were used in our literature searches: “assisted colonization,” “assisted migration,” “translocation,” “genetic rescue,” “evolutionary rescue,” “transgenerational plasticity,” “hybridi*ation AND outbreeding,” “hybridi*ation OR hybrid AND viability AND conservation”; (January 2018 using Web of Science or Google Scholar). The type of conservation intervention (i.e., the conservation “strategy”) was gleaned from study abstracts. One person (E. Crispo) performed this task for all studies included in the database. “Transgenerational plasticity” was considered a process‐related strategy that aims to alter phenotypes over generations in conjunction with environmental changes through acclimation instead of through evolution. “Genetic rescue” was considered a state‐related strategy where increasing genetic variation and population size was the goal, with no mention of adaptation to changing environments. “Evolutionary rescue” was similar as genetic rescue, but this term was used to refer to process‐related strategies that (a) specifically mentioned adaptation to changing environmental conditions over time, or (b) specifically referred to crosses between genetically or phenotypically distinct populations or ecotypes. Even if authors referred to genetic rescue in their papers, we called these strategies “evolutionary rescue” if the goal of promoting adaptation to changing environmental conditions specifically applied. Interspecific “hybridization” is a process because it is not possible to conserve the current state when crossing two species. “Demographic rescue” is certainly a state‐based strategy in studies that only mentioned increasing numbers, without mention of enhancing genetic variation. We included translocations and assisted colonization under “demographic rescue” when there was no implication of enhancing genetic diversity or evolutionary potential, and when movement of individuals was clearly intended only to improve survival.

After sorting through papers, a total of 15 articles on a total of 15 species were selected based on the four criteria (Supporting Information Table S1). The species covered a wide taxonomic breadth, including yeast, plants, invertebrate animals, and vertebrate animals. A total of 95 entries were included, with multiple entries from most studies. Some pseudoreplication occurred, when multiple experimental lineages were compared to the same control or starting population; however, we felt that this approach was inevitable for inclusion of the maximal amount of usable data. Most studies were on experimental populations, and only four entries were obtained from in situ conservation scenarios. Most entries tested evolutionary rescue (59 entries), followed by transgenerational plasticity (12 entries), genetic rescue (11 entries), interspecific hybridization (8 entries), and demographic rescue (5 entries). A majority of the studies measured survival (55 entries), followed by fecundity or related measures (32 entries), and abundance or recruitment (8 entries). We also recorded whether the experimental conditions imposed were stressful (40 entries, experimental stress treatment, e.g., high salt or heat shock) or benign (55 entries, control treatment, e.g., low salt or no heat shock, or no implicit stress treatment imposed).

Using the larger dataset that included studies without sampling variability or sample size, we were table to extract data from 35 studies on a total of 33 species (Supporting Information Table S2). This dataset included all 95 entries from the smaller dataset described above, and an additional 35 entries, for a grand total of 130 entries. For each entry, we recorded whether the longer time period increased or decreased fitness relative to the short time period, using the equation:

$$\left(w_{2}\;-\;w_{0}\;\text{or}\;w_{\text{control2}}\right)\;-\;\left(w_{1}\;-\;w_{0}\;\text{or}\;w_{\text{control1}}\right)\;=\;\text{difference}.$$

This difference was recorded as “positive,” “negative,” or 0. This was done for each of the 130 entries. We then created a collapsed database, with only one entry per combination of study, species, and conservation strategy (noting that some studies included multiple species or strategies). If all entries for a study or species or strategy had differences (as above) that were positive, we assigned that entry as “positive,” and similarly for differences that were all “negative.” If some entries were positive and others were negative for a given study/species/strategy, we assigned that entry a 0, meaning we were unable to conclusively determine whether the conservation implementation was beneficial over the long term.

### 3.2 Quantitative synthesis

We combined relative and absolute fitness in our assessment of the maladaptation‐fitness consequences for each of the five conservation strategies associated with conservation goals of adaptive state versus adaptive process (Table 2). For the dataset with 95 entries meeting all four criteria, we used the *metafor* R package (Viechtbauer, 2010) to estimate effect sizes for absolute and relative fitness as the standardized mean difference (SMD) and sampling variability for each of two time periods: (a) immediately after conservation intervention minus prior to conservation or the control and (b) two or more generations after conservation intervention minus prior to conservation or the control. For (a), we refer to the effect size as *y*_A_ and the sampling variance as *v*_A_ hereafter, and for (b), we refer to the effect size as *y*_B_ and the sampling variance as *v*_B_. We then estimated an overall effect size for the difference in fitness between these two time periods. To calculate pooled standard deviation for the overall effect sizes, we used the square root of *v*_A_ and *v*_B_. For the sample sizes to calculate pooled standard deviation for the overall effect sizes, we used the average *n* used for *v*_A_ and for *v*_B_, respectively. We performed analyses on this overall effect size using mixed‐model analyses in R (*metafor* package).

**Table 2:** Mean effect size (standardized mean difference) for fitness for each of the five conservation strategies

Strategy	*y*_A_	*y*_B_	*y*_B‐A_	Mean sampling variance of *y*_B‐A_
Transgenerational plasticity	0.1549667	0.4592083	0.5743917	0.1981333
Demographic rescue	−2.3910800	−2.2469400	0.9804200	0.1147000
Genetic rescue	0.3409182	0.3868636	0.2705545	0.2131909
Evolutionary rescue	0.6512169	0.7292898	0.3743068	0.3138407
Interspecific hybridization	0.9707125	−4.3995000	−3.0813250	1.6431000

Note

*y*_A_ is the effect size for absolute or relative fitness immediately after conservation intervention. *y*_B_ is the effect size for absolute or relative fitness at the last generation during which fitness was measured. *y*_B‐A_ is the effect size for the difference between *y*_B_ and *y*_A_. For the pooled standard deviation for *y*_B‐A_, we used the square root of the sampling variances for *y*_A_ and *y*_B_. For the sample sizes for *y*_B‐A_, we used the average for the sample sizes used to generate *y*_A_ and *y*_B_, respectively. Negative values for *y*_A_ indicate that the effect of conservation intervention was initially detrimental immediately after conservation, and positive values indicate it was beneficial. Negative values for *y*_B_ indicate that the conservation effect remained detrimental after multiple generations, whereas positive values of *y*_B_ indicated that the effect of conservation was positive after multiple generations. Negative values for *y*_B‐A_ indicate that, regardless of how conservation impacted fitness, fitness decreased across the generations, whereas positive values of *y*_B‐A_ indicated that fitness increased relative to fitness immediately after conservation.

Mixed‐model analyses were performed using the rma function in *metafor*, and the response variable included the overall effect size as described above (Supporting Information Table S3). The moderators included were conservation strategy (5 levels), species (15 levels), and type of fitness measure (3 levels); we also included the maximum number of generations as a covariate. Next, we performed sensitivity analyses within each of the five conservation strategies separately, using the leave1out function in R to determine the impact of individual entries on estimates of effect sizes within conservation strategies (Supporting Information Table S4). We followed this analysis of effect sizes with analyses on the larger dataset that included studies for which sampling variability and sample size were not available. We performed two separate contingency tests for associations between two categorical variables (Table 3): conservation strategy (5 levels) and fitness outcome (positive, negative, no change). We did this for all 130 entries (without controlling for the fact that we had multiple entries per study and per species) and including only one value per combination of study, species, and conservation strategy (39 entries).

**Table 3:** Categorical data used for contingency tests to assess whether conservation strategy resulted in increased or decreased fitness across time

Strategy	All entries			One entry per study/species/strategy		
	Increase	Decrease	No change	Increase	Decrease	No change
Demographic rescue	7	7	2	4	2	3
Genetic rescue	25	8	0	9	1	4
Transgenerational plasticity	6	4	0	0	0	2
Evolutionary rescue	24	26	1	2	0	3
Interspecific hybridization	6	14	0	1	5	3

Note

Two data sets were analyzed: one which included all entries and did not control for multiple entries per study and species (total 130 entries) and one which included only a single entry per combination of study, species, and strategy (total 39 entries).

## 4 RESULTS

**Figure 2:**
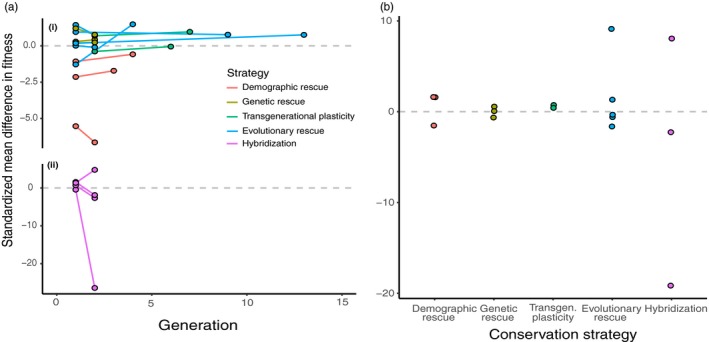
Fitness responses to different conservation strategies. (a) Standardized mean differences (SMD) were calculated for fitness values measured over three time periods (1. before conservation, 2. soon after conservation, and 3. multiple generations after conservation) (Table 3). SMDs between time periods 1–2 and 2–3 are shown with respect to generation time. Because of differences in magnitude, SMDs for (ii) “hybridization” are shown separately (and using a different scale) from SMDs pertaining to (i) all other strategies. (b) The SMD was also calculated between each of these two time periods to evaluate the overall effect of each conservation strategy

Most conservation strategies had positive but weak effects on population fitness across generations (Table 2, *y*_B‐A_ < 1; Figure 2). Effect sizes for demographic rescue, genetic rescue, evolutionary rescue, and transgenerational plasticity ranged from 0.27 to 0.98 and had 95% CIs that included 0 (Table 2). Demographic rescue reduced fitness immediately after conservation invention and remained detrimental after multiple generations (*y*_A_ and *y*_B_, Table 3), regardless of whether abundance or survival was used as the fitness measure. Over time, the negative fitness effect was alleviated, resulting in a positive effect size for change in fitness, even though populations remained maladapted (*y*_A‐B_; Table 2). For transgenerational plasticity, genetic rescue, and evolutionary rescue, mildly positive effects occurred immediately after conservation intervention, after several generations, and across generations (Table 2). However, the exception was interspecific hybridization, which had an overall negative impact on population fitness (SMD *y*_A‐B_ = −3.08; Table 2). There was an initial increase in fitness in the F_1_ generation (*y*_A_, Table 2), followed by a dramatic reduction in fitness after several generations and across generations following hybridization (*y*_B_, *y*_A‐B_; Table 2). Compared to the other conservation strategies, this effect was relatively strong but also had a 95% CI that included 0 (Figure 2). Standardized mean difference (SMD) in fitness across time periods varied with respect to conservation strategy, type of fitness measure, and species (all *p* < 0.05; Supporting Information Table S3). In separate analyses of each conservation strategy, we found no significant moderators for transgenerational plasticity, demographic rescue, and genetic rescue. However, for evolutionary rescue, all three moderators (i.e., species, fitness type, and generations) were significant. For interspecific hybridization, only species was a significant moderator.

Contingency tests showed that conservation strategies differed with respect to whether they improved fitness over time, decreased fitness over time, or had no impact on fitness (Table 3). This was the case regardless of whether we analyzed the entire data set without controlling for variation among studies or species, and when analyzing a single value per combination of study, species, and conservation strategy. In both cases, hybridization resulted in more negative effect sizes than expected, and genetic rescue resulted in more positive effect sizes than expected. Individual entries did not impact estimates of effect sizes within conservation strategies (Supporting Information Table S4).

## 5 DISCUSSION

Our study based on a very small number of 15 studies indicated that success of each conservation strategy varies greatly across study systems. A general trend was that no single conservation strategy was especially effective at increasing fitness, either shortly after implementation or many generations later (Figure 2). In fact, many studies showed an initial decrease in fitness immediately after conservation. Indeed, all demographic rescue studies showed an initial decline in fitness, which persisted after several generations but was alleviated over time across generations (Table 2). This decline was the case regardless of whether abundance or survival was used as the fitness measure, a surprising result given that the goal of demographic rescue is to increase abundance. For all other conservation strategies, average effect sizes for fitness were initially positive despite individual study variation in effect size (Supporting Information Table S5). This result suggests that the nuances of individual study systems are more important to consider than overall conservation strategy that is to be employed.

Interspecific hybridization was the most variable strategy, resulting in the most extreme positive and negative fitness effects. Thus, interspecific hybridization as a strategy might be considered risky but also potentially transformative. For instance, only one study showed positive effects of interspecific hybridization over the long term (from experimental evolution of salt‐stressed yeast). The other two studies, using oysters and amphibians in benign environments, showed negative effects of hybridization over the long term. One explanation is that outbreeding depression only manifests in F_2_ generations or later, for instance due to recombination leading to the breakdown of coadapted gene complexes (Frankham, 2015; Tallmon et al., 2004). Speculatively, interspecific hybridization might be most beneficial in extreme environments where adaptation via standing variation would otherwise not be possible. On average, interspecific hybridization leads to the largest decrease in fitness over time, despite an initially positive effect size immediately after conservation implementation (Table 2) suggesting extreme caution should be used when considering to employ this strategy.

While fitness tended to increase over time across strategies other than interspecific hybridization (Table 2; i.e., positive slopes in Figure 2a), slopes were generally low. Moreover, all strategies other than transgenerational plasticity included both positive and negative slopes (Figure 2b). One possible explanation for this prevalence of fitness declines followed by generally weak increases is that conservation strategies might require many generations to yield substantial gains in fitness. Thus, it is not necessarily surprising that fitness commonly declines shortly after conservation implementation. On the other hand, it is surprising to consider that taking no conservation action in some cases action might have resulted in higher fitness than was achieved through an implementation strategy. An alternative explanation is that fitness declines, as would be depicted as even more negative or less positive slopes than those we observed, would have been documented without any conservation intervention.

## 6 CONCLUSION

Identifying where maladaptation is happening is a major challenge for the conservation of populations and species. This is because an inevitable trade‐off often exists between adaptive state and adaptive process goals in many conservation contexts. For example, demographic rescue programs are often implemented to rescue populations from extinction, and so maladaptation that immediately affects survival and individual reproductive success may be more of a priority than longer‐term adaptive process—that is, phenotypic matching and contemporary selection help to ensure population persistence now, as opposed to a potential, unforeseen risk to the population in a future context that may or may not happen should phenotypic/genetic variance be lacking. In our meta‐analysis, most conservation strategies that spanned the spectrum of conservation goals from adaptive state to adaptive process had positive but weak effects on population fitness over generations, and they slowed the rate of population fitness decline. However, the exception was interspecific hybridization, where the effects of conservation intervention on population fitness were case dependent and highly variable following the F_1_ generation.

We are still only becoming more aware of the myriad ways in which (mal)adaptation is generated by human‐induced environmental changes and how these might affect species/population persistence. New tools are available that can enhance our ability to detect and manage maladaptation. These include CRISPR genome editing, transgenerational acclimation, targeted management of reduce negative effects of size‐selective harvest, and progress on manipulating captive breeding programs (e.g., fish culture) to minimize phenotype–environment mismatch in postrelease habitat. For example, using these tools, maladaptation could be quantified in small populations as to determine whether interventions are needed: Are we dealing with a small, declining population where intervention could increase or decrease the likelihood of population persistence? Does the nature of the maladaptation and situation (species, generation time, habitat) require immediate intervention, more careful follow‐up monitoring, both, or leaving things alone? Decision‐making must carefully consider such trade‐offs at any time point during the implementation or continuation of conservation strategies.

## CONFLICT OF INTEREST

None declared.

## Data Availability

Data for the meta‐analysis (Supporting Information Tables S1 and S2) are archived with Dryad: https://doi.org/10.5061/dryad.76dc375
