# Supplementary material for: Conservation through the lens of (mal)adaptation: Concepts and meta‐analysis
Source: Evol Appl. 2019 Apr 6;12(7):1287–304. doi: 10.1111/eva.12791 (PMC6691223; doi:10.1111/eva.12791)
Supplement: Supplementary file 3 [file EVA-12-1287-s003.docx]

**Table S3.** Results from the mixed-effects model used to analyze moderator effects on the change in relative or absolute fitness between two time periods: immediately after conservation intervention versus a later generation. “Strategy” included five levels (transgenerational plasticity, demographic rescue, genetic rescue, evolutionary rescue, and interspecific hybridization). “Fitness” included three levels of fitness measures (survival, fecundity, and abundance/population size/recruitment). There were a total of 15 species analyzed in 15 separate studies (with 1 study examining 2 species, and 2 species studied in 2 separate studies). “Generation” was a numerical covariate that included the maximum number of generations for each entry. The estimated amount of residual heterogeneity (tau^2^) was 8.57 (SE=1.33), the residual heterogeneity (I^2^) was 98.4%, the unaccounted variability (H^2^) was 64.27, and the R^2^ was 24.47%. *significant at the 0.05 level. **significant at the 0.01 level. ***significant at the 0.001 level.

| Moderator | 95% CI lower bound | Estimate of effect | 95% CI upper bound |
| --- | --- | --- | --- |
| Conservation strategy | 0.2313 | 1.0104* | 1.7896 |
| Species | 0.3606 | 0.6947*** | 1.0288 |
| Type of fitness measure | -2.6033 | -1.6155** | -0.6276 |
| Maximum generations | -0.4501 | -0.2040 | 0.0421 |
