# Supplementary material for: Conservation through the lens of (mal)adaptation: Concepts and meta‐analysis
Source: Evol Appl. 2019 Apr 6;12(7):1287–304. doi: 10.1111/eva.12791 (PMC6691223; doi:10.1111/eva.12791)
Supplement: Supplementary file 4 [file EVA-12-1287-s004.docx]

**Table S1.** The effects of individual entries on the effect sizes for the difference in relative or absolute fitness between the two time periods we used the “leave 1 out” function in the *metafor* package in R. We analyzed each conservation strategy separately. The range in effect sizes were positive for each strategy except hybridization, for which the range was negative. This result indicates that individual entries had no effect on our overall conclusions regarding the different conservation strategies’ effects on fitness over time.

| Conservation strategy | Minimum mean effect size | Maximum mean effect size |
| --- | --- | --- |
| Transgenerational plasticity | 0.3571 | 0.7968 |
| Demographic rescue | 0.1341 | 1.5812 |
| Genetic rescue | 0.0302 | 0.5372 |
| Evolutionary rescue | 0.1417 | 0.4484 |
| Interspecific hybridization | -4.6661 | -0.6957 |
