# Supplementary material for: Conservation through the lens of (mal)adaptation: Concepts and meta‐analysis
Source: Evol Appl. 2019 Apr 6;12(7):1287–304. doi: 10.1111/eva.12791 (PMC6691223; doi:10.1111/eva.12791)
Supplement: Supplementary file 5 [file EVA-12-1287-s005.docx]

**Supplemental Information**

**Table S5. Forest plots of fitness effects for each of five different conservation strategies.** Each plot shows the studies analyzed for a given conservation strategy. For each study, standardized mean difference (SMD) was calculated between yA and yB, each of which is also a standardized mean difference. Specifically, yA was calculated as the standardized mean difference in fitness immediately after versus immediately before conservation action (or compared to a control group). yB was calculated as the standardized mean difference in fitness two or more generations after conservation action versus fitness before conservation action (or a control group). In each plot, the overall effect (with 95% CI) is estimated from a random effects model meta-analysis that included only the studies shown in the plot (i.e. corresponding to a given conservation strategy).
